# Voltage-gated sodium channel expression and action potential generation in differentiated NG108-15 cells

**DOI:** 10.1186/1471-2202-13-129

**Published:** 2012-10-25

**Authors:** Jinxu Liu, Huiyin Tu, Dongze Zhang, Hong Zheng, Yu-Long Li

**Affiliations:** 1 Department of Emergency Medicine, University of Nebraska Medical Center, Omaha, NE, 68198, USA; 2 Department of Cellular & Integrative Physiology, University of Nebraska Medical Center, Omaha, NE, 68198, USA

**Keywords:** Action potential, Na^+^ channel, NG108-15 cell, Patch clamp, Single-cell real-time PCR, Western blot

## Abstract

**Background:**

The generation of action potential is required for stimulus-evoked neurotransmitter release in most neurons. Although various voltage-gated ion channels are involved in action potential production, the initiation of the action potential is mainly mediated by voltage-gated Na^+^ channels. In the present study, differentiation-induced changes of mRNA and protein expression of Na^+^ channels, Na^+^ currents, and cell membrane excitability were investigated in NG108-15 cells.

**Results:**

Whole-cell patch-clamp results showed that differentiation (9 days) didn’t change cell membrane excitability, compared to undifferentiated state. But differentiation (21 days) induced the action potential generation in 45.5% of NG108-15 cells (25/55 cells). In 9-day-differentiated cells, Na^+^ currents were mildly increased, which was also found in 21-day differentiated cells without action potential. In 21-day differentiated cells with action potential, Na^+^ currents were significantly enhanced. Western blot data showed that the expression of Na^+^ channels was increased with differentiated-time dependent manner. Single-cell real-time PCR data demonstrated that the expression of Na^+^ channel mRNA was increased by 21 days of differentiation in NG108-15 cells. More importantly, the mRNA level of Na^+^ channels in cells with action potential was higher than that in cells without action potential.

**Conclusion:**

Differentiation induces expression of voltage-gated Na^+^ channels and action potential generation in NG108-15 cells. A high level of the Na^+^ channel density is required for differentiation-triggered action potential generation.

## Background

Exploring cell molecular and electrophysiological properties such as expression and current of ion channels, and action potentials is very important for understanding the physiological and pathophysiological functions of the excitable cells including neurons, muscle cells, and endocrine cells. Although acute-isolated primary cell is the optimum choice for pursuing these measurements, cell lines are also served as an appropriate tool for the cell molecular and electrophysiological studies, because cell lines provide the advantage of enough homogeneous cells that can make the investigation under easily controlled conditions.

NG108-15 cell line is a hybrid cell line formed by the fusion of mouse N18TG2 neuroblastoma cells and rat C6-BU-1 glioma cells [1]. After differentiation, this cell line presents neurite extension, forms synapses, and develops the ultimate neural property of acetylcholine release and specific activities of choline acetyltransferase and acetylcholinesterase [2-4]. Therefore, many studies used NG108-15 cells as the cholinergic cells to investigating electrophysiological kinetics and cell functions of neurons [4-12].

Action potential is an important physiological feature of the excitable cells. In most vertebrate neurons, action potential production is required for neuronal excitation and stimulus-evoked neurotransmitter release, which are involved in neuron-to-neuron communication [13,14]. Although action potentials are generated by voltage-gated Na^+^, K^+^, and Ca^++^ channels existed in the cell membrane, the influx of Na^+^ ions through voltage-gated Na^+^ channels plays the most important role in the initiation and propagation of action potential [15,16]. In order to clarify the relationship between action potential generation and sodium channel density, in this study, we investigated the time-course for differentiation-induced changes of membrane excitability and Na^+^ channels in NG108-15 cells.

## Results

### Differentiated NG108-15 cells bear cholinergic neuronal property

**Figure 1:**
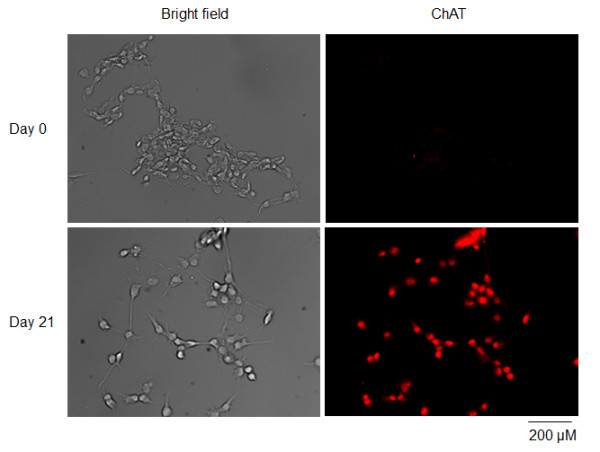
Expression of choline acetyltransferase (ChAT, a cholinergic neuronal marker) in NG108-15 cells at undifferentiated state or after 21 days of differentiation, measured by immunofluorescence staining.

As a cholinergic neuron marker, choline acetyltransferase (ChAT) was detected in NG108-15 cells by immunofluorescence staining. In the undifferentiated cells, there were a few ChAT-positive cells and very low-level ChAT images in the ChAT-positive cells (Figure 1). After 21 days of differentiation, all cells were ChAT positive and presented high-level ChAT images (Figure 1).

### Differentiation-induced action potential in NG108-15 cells

**Figure 2:**
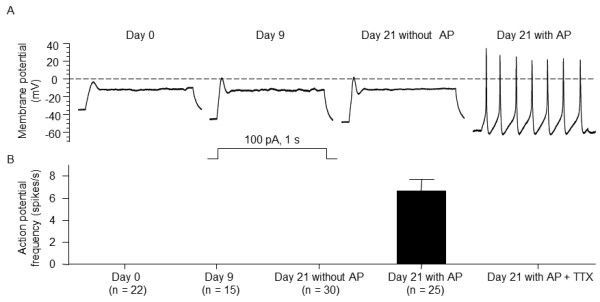
**Alterations in membrane excitability of NG108-15 cells induced by differentiation.A**: Representative traces show the membrane potential response to a depolarizing current injection (100 pA, 1 sec) under whole-cell current-clamp configuration. **B**: Action potential (AP) frequency in undifferentiated (day0) and differentiated NG108-15 cells. TTX: tetrodotoxin. Data are means ± SEM, n is the number of cells.

Using whole-cell current-clamp recording method, the response of cell membrane to current injections was measured in undifferentiated and differentiated NG108-15 cells (Figure 2 and Table 1). In undifferentiated cells (day0), the resting membrane potential (RMP) was −34.9 ± 1.4 mV (n=22), and current injection (100 pA) induced a small depolarization. However, the action potential didn’t appear in undifferentiated cells.

**Table 1:** Differentiation-induced changes in membrane properties of NG108-15 cells

		**RMP**	**V**_**max**_	**Depolarization amplitude**	**Current threshold**
	**n**	**(mv)**	**(mV/ms)**	**(mV)**	**(pA)**
Day 0	22	−34.9 ± 1.4	20.1 ± 0.9	32.1 ± 1.6	n/a
Day 9	15	−43.2 ± 1.5*	23.2 ± 1.2	45.0 ± 2.9*	n/a
Day 21					
Without AP	30	−45.4 ± 2.1*	28.5 ± 3.5*	46.1 ± 4.7*	n/a
Day 21					
with AP	25	−56.1 ± 1.6*^#$^	136.1 ± 13.7*^#$^	79.4 ± 7.4*^#$^	48.2 ± 2.7

*RMP* resting membrane potential; *V*_*max*_ maximum rate of depolarization of action potential (AP). Data are means ± SEM, n is the number of cells studied. *P < 0.05 vs. 0 day of differentiation (day 0, undifferentiated cells); ^#^P < 0.05 vs. cells after 9 days of differentiation (day 9); ^$^P < 0.05 vs. cells after 21 days of differentiation (day 21) without AP.

After 9 days of differentiation, the RMP was increased to −43.2 ± 1.5 mV (n= 15; P < 0.05 vs. Day 0), and the depolarization amplitude was increased to 45 ± 2.9 mV (P < 0.05 vs. Day 0), compared with those in undifferentiated state. However, current injection (100 pA) didn’t yet induce the action potential in the cells with 9 days of differentiation.

After 21 days of differentiation, 45.5% of cells (25/55) had the ability to generate the action potential upon the current injection (100 pA), and the average frequency of action potentials was 6.7 ± 1.1 spikes/s (Figure 2). A Na^+^ channel antagonist tetrodotoxin (TTX, 1μM) could totally block the generation of action potential, indicating that differentiation-induced action potential is Na^+^ channel dependent. The current threshold-inducing action potential was 48.2 ± 2.7 pA in the cells with action potential. We also found that the cells with action potential had a more negative RMP of −56.1 ± 1.6 mV, compared to day 0, day 9, and day 21 without action potential cells (Table 1). On the contrary, the other cells (54.5%, 30/55) didn’t generate the action potential after 21 days of differentiation. The cells without action potential presented similar electrical characteristics to those cells with 9 days of differentiation (Table 1).

Additionally, we also measured the generation of the action potential after 35 days of differentiation. After 35 days of differentiation, there was no significant difference on the ratio of cells with/without action potential (data not shown), compared with that after 21 days of differentiation.

### Changes of Na^+^ currents induced by differentiation

**Figure 3:**
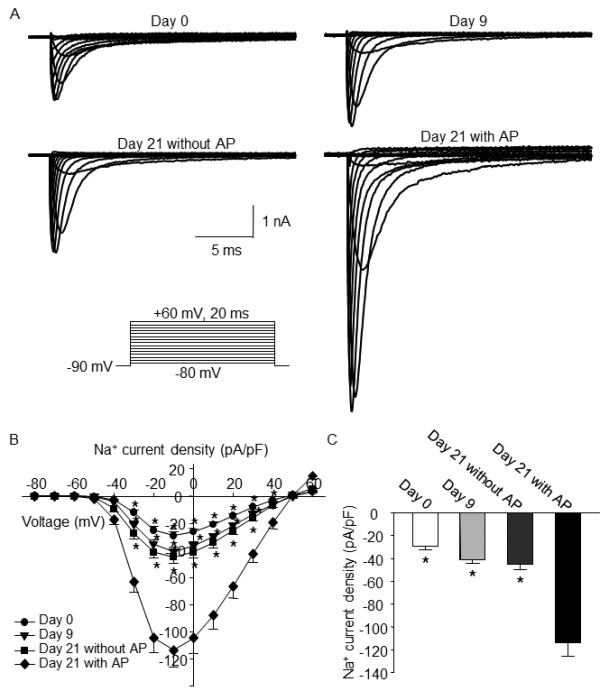
**Original recording (A), current–voltage (I-V) curve (B), and mean data (C) of the voltage-gated Na**^**+**^**currents in undifferentiated and differentiated NG108-15 cells, measured by whole-cell voltage-clamp technique.** Data are means ± SEM, n = 10 cells in each group. *P < 0.05 vs. cells after 21days of differentiation with action potential (AP).

After the recording of the action potential, Na^+^ currents were recorded under voltage-clamp mode. Using this method, the Na^+^ currents and action potential were recorded in the same cell. In the undifferentiated cells, the Na^+^ current density was −29.2 ± 3 pA/pF (n= 10). After 9 days of differentiation, the Na^+^ current density of NG108-15 cells increased to −40.8 ± 3.3 pA/pF (n=10, p > 0.05 vs. undifferentiated cells). After 21 days of differentiation, the Na^+^ current density was not significantly altered in the cells without action potential (−44.8 ± 4.7 pA/pF; n=10), compared to that in the cells with 9 days of differentiation. However, the Na^+^ current density was significantly increased in the cells with action potential (−113.5 ± 12.4 pA/pF, n=10; p < 0.05 vs. day 0, day 9, and day 21 without action potential cells; Figure 3). The Na^+^ current density detected in undifferentiated and differentiated cells was completely inhibited by 1 μM TTX (data not shown), suggesting that only TTX-sensitive Na^+^ channels exist in NG108-15 cells.

### Expression of Na^+^ channel protein induced by differentiation

**Figure 4:**
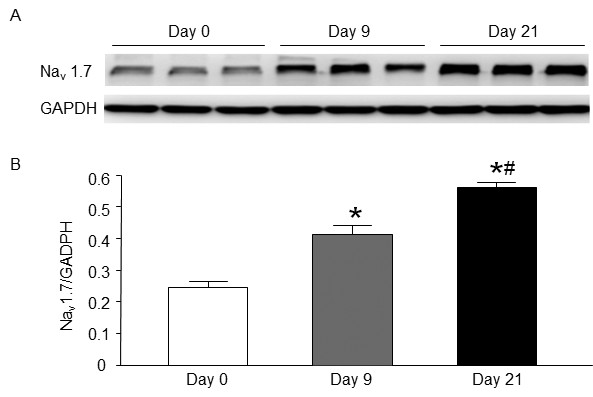
**Representative (A) and summary (B) data for the protein expression of Na**_**v**_**1.7 in undifferentiated and differentiated NG108-15 cells, measured by western blot analysis.** Data are means ± SEM, n = 3 in each time-point. *P < 0.05 vs. 0 day of differentiation (undifferentiated cells), ^#^P < 0.05 vs. 9 days of differentiation.

It has been reported that only Na_v_1.7 (a primary TTX-sensitive Na^+^ channels) expression is increased after NG108-15 cell differentiation [9]. Therefore, we used western blot analysis to measure the expression of Na_v_1.7 protein in undifferentiated and differentiated NG108-15 cells. As shown in Figure 4, the protein level of Na_v_1.7 increased after 9 days of differentiation and further enhanced after 21 days of differentiation. We couldn’t compare the expression of Na_v_1.7 protein between the cells with and without action potential due to technical limitation.

### Expression of Na_v_1.7 mRNA induced by differentiation

**Figure 5:**
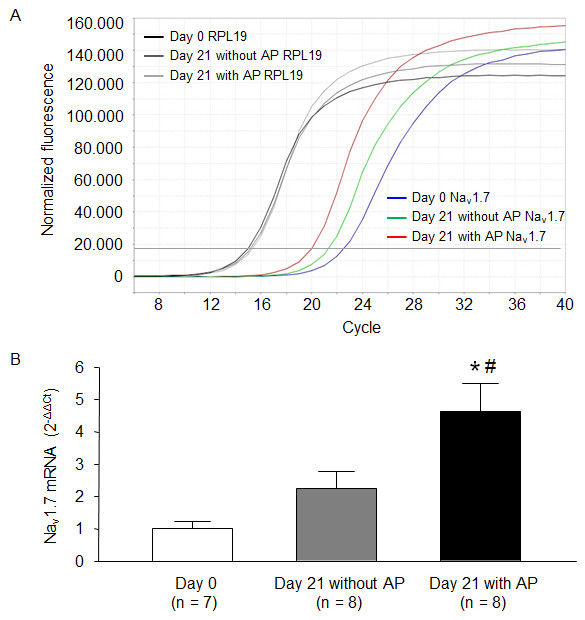
**Representative (A) and summary (B) data for the mRNA expression of Na**_**v**_**1.7 in undifferentiated and differentiated NG108-15 cells; quantified by single-cell real-time PCR, RPL19 (a housekeeping gene) was used as the internal control.** Data are means ± SEM, n is the number of cells. *P < 0.05 vs. 0 day of differentiation (undifferentiated cells), ^#^P < 0.05 vs. cells after 21 days of differentiation without AP.

Using single-cell real-time PCR, we measured the Na_v_1.7 mRNA in undifferentiated and differentiated NG108-15 cells. After 21 days of differentiation, expression of Na_v_1.7 mRNA significantly increased in NG108-15 cells. More importantly, the level of Na_v_1.7 mRNA in the cells with action potential was higher than that in the cells without action potential (Figure 5).

### Relationships among Na_v_1.7 mRNA, Na^+^ current density, and action potential frequency

**Figure 6:**
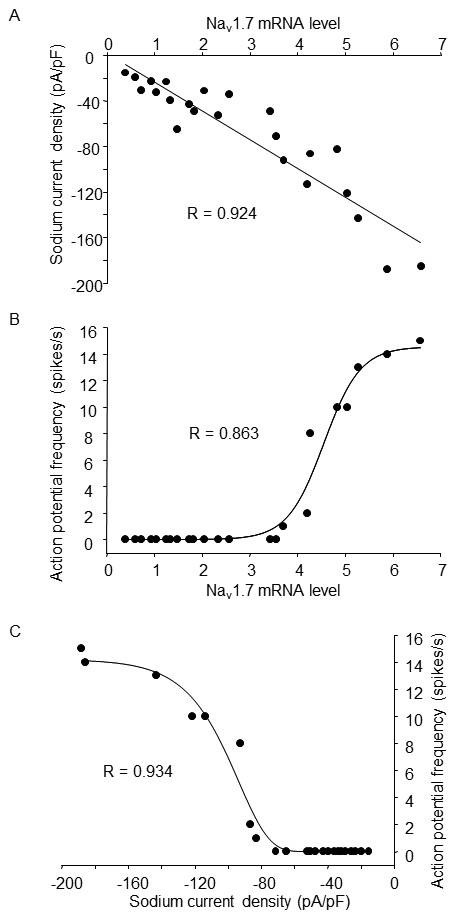
**Correlation analyses among Na**_**v**_**1.7 mRNA, Na**^**+**^**current density, and action potential frequency in NG108-15 cells.** N=23 cells including 7 undifferentiated cells and 16 cells after 21-day differentiation without or with action potential. R is the correlation coefficient.

Using linear regression analysis, we found that there were correlations among Na_v_1.7 mRNA, Na^+^ current density, and action potential frequency (Figure 6). Additionally, we also determined that 77 pA/pF (Na^+^ current density) was required to induce the generation of an action potential in NG108-15 cells.

## Discussion

Previous studies have found that during the differentiation of NG108-15 cells, dibutyryl cyclic AMP is a key factor in the culture medium, which can stimulate NG108-15 cells to present a morphological alterations (such as the increase in cell diameter, neurite length, and number of clear vesicles) and to develop the cholinergic neuronal properties including stimulus-dependent acetylcholine release and activities of ChAT and acetylcholinesterase [2-4]. Our present study demonstrated that ChAT, a cholinergic neuron marker, was expressed in all differentiated NG108-15 cells (Figure 1). Based on these results, NG108-15 cell line is considered to be a suitable cell line for studying cholinergic neuronal function.

Although many studies focused on the measurement of ion channels (such as voltage-gated Na^+^, Ca^++^, and K^+^ channels) in NG108-15 cells [5-10,17-20], a few studies recorded the action potential and obtained inconsistent results [21-23]. In Kowtha’s study, the differentiated-cell excitability is still lower under a very high current-stimulation (30 nA) [22]. Extracellularly added NH_4_Cl increased the cell excitability via an elevation in intracellular pH [22]. Doebler reported that a high current-stimulation (700 pA, 75 ms) induced the generation of action potential after NG108-15 cells were differentiated over 5 days [21]. Usually, action potential recording needs to be kept a long time for measuring the cell excitability (including action potential frequency and current threshold-inducing action potential) and investigating the effect of drugs. However, the high current-stimulation can induce the cell damage and shorten the cell recording time. Additionally, a current stimulation less than 300 pA is normally used for action potential recording in isolated primary neuron cells. In present study, therefore, we chose a low current stimulation (100 pA) to investigate the time course for differentiation-induced alteration of cell excitability in NG108-15 cells. We observed that short-time differentiation (9 days) didn’t change the cell excitability, compared with undifferentiated condition (Figure 2). Moreover, only about a half of cells generated action potentials after 3 week differentiation (Figure 2). A similar result was found in Ma’s study [23]. This research group considered there are two types of the cells after long-time differentiation: type 1 neuron-like cells with neuronal morphologies and excitable membrane properties, and type 2 cells with a proliferative property [23].

It is well known that Na^+^ currents mainly mediate the upstroke of action potential [15,24]. However, it is unclear how much functional Na^+^ channel and Na^+^ current density are required for the generation of neuronal action potential in NG108-15 cells. We recorded the action potential and voltage-gated Na^+^ currents in the same cell. Using this technique, we found that differentiation-induced mild increase of Na^+^ currents didn’t trigger the generation of action potential (such as day 9 and day 21 without AP groups, Figure 3), and a significant increase of Na^+^ currents is needed for generating action potentials in differentiated NG108-15 cells (such as day 21 with AP group, Figure 3). The data from linear regression analysis (Figure 6) suggest that differentiation-enhanced Na^+^ current density should reach a high level (77 pA/pF) close to the current threshold for generating action potentials in NG108-15 cells, which is also supported by western blot and single-cell real-time PCR data (Figures 4 and 5). Similarly, a previous study has demonstrated that action potential generation requires a high Na^+^ channel density in the axon initial segment of cortical layer 5 pyramidal neurons [24].

There is no direct evidence showing the involvement of Na_v_ channels in acetylcholine release from cholinergic neurons. However, many studies have shown that Ca^++^ influx through the voltage-gated Ca^++^ channels is a key trigger for the release of neurotransmitters including acetylcholine [25-31]. Voltage-gated Ca^++^ channels are activated and intracellular Ca^++^ level is increased when the cell membrane is depolarized by an action potential [32-34]. Therefore, Na^+^ channel-initiated action potential might link to the acetylcholine release through triggering Ca^++^ influx.

Our recent study has shown that differentiation also induces the alteration of voltage-gated Ca^++^ channel mRNA, protein and current in NG108-15 cells [10]. Additionally, 0.1 mM Cd^++^ (a common voltage-gated Ca^++^ channel blocker) reduces the action potential frequency and increases the current threshold-inducing action potential in differentiated NG108-15 cells (data not shown). It is possible that enhanced expression of voltage-gated Ca^++^ channels also contributes to action potential generation in NG108-15 cells.

In addition to fasting activating and inactivating Na^+^ current, the persistent Na^+^ current, also known as non-inactivating Na^+^ current are recorded in many types of excitable neurons [35,36]. Wu, et al. reported that the non-inactivating Na^+^ current was characterized in differentiated NG108-15 cells, and this type of Na^+^ current might facilitate neuronal hyper-excitability [37]. However, the origin of the non-inactivating Na^+^ current is unclear. Three hypotheses have been proposed: first, the non-inactivating Na^+^ current originates from the window current; second, the non-inactivating Na^+^ current originates from the mutation in the inactivation properties of the same channels that generate the fasting activating and inactivating Na^+^ currents; third, the non-inactivating Na^+^ current originates from a special subtype of Na^+^ channels [35,36]. Based on these uncertainties, we did not address the correlation between non-inactivating Na^+^ current and action potential in the present study.

Although established neuronal cell lines including NG108-15 cells may provide some valuable data, extrapolation to the original neuronal cells should be cautiously used because an endless debate for the neuronal cell lines is still presented in respect of genetic, differentiated, biochemical, and physiological aspects.

## Conclusion

It is the first time to investigate the time course for differentiation-induced changes in cell excitability and voltage-gated Na^+^ channels in NG108-15 cells using electrophysiological and molecular techniques. Differentiation time-dependently enhances the expression and current density of voltage-gated Na^+^ channels and induces about a half of cells to generate action potential. These results suggest that action potential is generated in differentiated NG108-15 cells with a high voltage-gated Na^+^ channel density.

## Methods

### Cell culture and differentiation

The neuroblastoma × glioma NG108-15 cell line was obtained from the American Type Culture Collection (ATCC, Manassas, VA). NG108-15 cells were cultured as previously described [10]. Briefly, cells were cultured at a density of 1 × 10^4^ cells/cm^2^ on either a 60 mm plastic dish or a 35 mm plastic dish containing glass cover slips in Dulbecco’s modified Eagle’s medium (DMEM) with 10% fetal bovine serum. Differentiation was induced by culturing the cells in a serum-free medium consisting of DMEM, N2 supplements, 1 mM dibutyryl cyclic AMP, and antibiotics. Cells were used for experiments after 0–21 days of differentiation.

### Immunofluorescent staining

NG108-15 cells plated onto coverslips were fixed with 4% paraformaldehyde for 10 min at 4°C and then blocked with 10% normal goat serum for 1 h at room temperature. The NG108-15 cells were incubated with primary antibody against choline acetyltransferase (ChAT; Novus Biologicals, Littleton, CO) overnight at 4°C. Then, the NG108-15 cells were incubated with fluorescence-conjugated secondary antibody (Santa Cruz Biotechnology, Santa Cruz, CA) for 1 h at room temperature. Finally, the NG108-15 cells were observed under a Leica fluorescent microscope with corresponding filter. Pictures were captured by a digital camera system. No staining was seen when PBS was used instead of the primary antibody in the above procedure.

### Recording of action potentials and sodium currents

Action potential and Na^+^ currents were recorded by the whole-cell patch-clamp technique using Axonpatch 200B patch-clamp amplifier (Axon Instruments, Sunnyvale, CA).

Action potential was recorded in the current-clamp mode. Resistance of the patch pipette was 1–2 MΩ when filled with (in mM) 105 K-aspartate, 20 KCl, 1 CaCl_2_, 5 MgATP, 10 HEPES, 10 EGTA, and 25 glucose, pH 7.2 with KOH. RNase inhibitor was added to the pipette solution to prevent the degradation of mRNA. The extracellular solution consisted of (in mM): 140 NaCl, 5.4 KCl, 0.5 MgCl_2_, 2.5 CaCl_2_, 5.5 HEPES, 11 glucose, and 10 sucrose, pH 7.4 with NaOH. Action potential was elicited by a ramp current injection of 0–300 pA to measure the current threshold-inducing action potential. The clamp-current at generation of the first action potential is defined as the current threshold-inducing action potential. Frequency of action potentials was measured in a 1second current clamp (100 pA).

To record Na^+^ currents after action potential recording in the same cell, the extracellular solution was changed to a solution consisting of (in mM): 70 NaCl, 60 choline-Cl, 10 CsCl, 10 TEA-Cl, 2 4-AP, 0.1CdCl_2_, 4 MgCl_2_, 10 HEPES, and 10 glucose, pH 7.4 with CsOH. Junction potential was calculated to be +9.7 mV using pClamp 10.2 software, and all values of membrane potential given throughout were corrected using this value. Series resistance of 5–10 MΩ was electronically compensated 80-90%. Current traces were sampled at 10 kHz and filtered at 5 kHz. Na^+^ currents were evoked from a holding potential of −90 mV by stepping to voltages between −80 and +60 mV in 10 mV increment for 20 ms. 1 μM tetrodotoxin was used to block Na^+^ currents. Peak currents were measured for each test potential and current density was calculated by dividing peak current by cell membrane capacitance. pClamp 10.2 program (Axon Instruments) was used for data acquisition and analysis. All experiments were done at room temperature. After the recording of Na^+^ currents, the cell was also used for single-cell real-time RT-PCR experiments.

### Western blot

Western blot was performed as described previously [38]. The protein of NG108-15 cell lysates was extracted with the lysing buffer (10 mM Tris, 1 mM EDTA, 1% SDS, pH 7.4) plus protease inhibitor cocktail (Sigma-Aldrich, 100 μl/ml). Total protein concentration was determined using a bicinchoninic acid protein assay kit (Pierce, Rockford, IL). Equal amounts of the protein samples were loaded and then separated on a 10% sodium dodecyl sulfate (SDS)-polyacrylamide gel. The proteins of these samples were electrophoretically transferred to PVDF membrane. The membrane was probed with mouse anti-Na_v_1.7 antibody (NeuroMab, Davis, CA) and a peroxidase-conjugated goat anti-mouse secondary antibody (Pierce). The signal was detected using enhanced chemiluminescence substrate (Pierce) and the bands were analyzed using UVP bioimaging system. The membrane was reprobed with mouse anti-GAPDH antibody (Santa Cruz Biotechnology, Santa Cruz, CA) and normalizing target protein intensity to that of GAPDH.

### Single-cell real-time RT-PCR

Single-cell real-time RT-PCR was performed as described previously [10,39]. After the recording of Na^+^ currents, the cellular content was obtained by applying suction on patch pipette and expelled into a 0.2-ml PCR tube containing following reagents: 5 μl volume consisting of 1 μl 5X lysis buffer (100 μl 5X lysis buffer consisting of 20 μl 5X M-MLV reverse transcriptase buffer (Invitrogen, Carlsbad, CA), 5 μl Nonidet P-40, 75 μl RNase-free water), 0.5 μl RNA guard Mix (100 μl consisting of 20 μl 5X M-MLV reverse transcriptase buffer, 20 μl RNase inhibitor, 60 μl RNase-free water), and 3.5 μl RNase-free water, and kept at −80°C until reverse transcription (RT) was performed.

After thawing was completed, the content of each tube (5 μl) was added to the PCR reaction containing 4 μl iScript Reaction Mix (Bio-Rad, Hercules, CA), 1 μl iScript Reverse Transcriptase (Bio-Rad), and 10 μl RNase-free water and then was reversely transcribed at 42°C for 30 min. The cDNA was then stored at −80°C.

There were two rounds of amplification for PCR. The sequences of the primers used in the present study were as follows. Na_v_1.7 (Genbank accession number NM_018852) forward: 5^′^-GTGGTGTCGCTTGTTGATGG-3^′^, reverse: 5^′^-CCTTTGCCTGAGATGTGGGT-3^′^, internal: 5^′^-CCCCAATGGACAGCTTCTTC-3^′^; RPL19 (Genbank accession number NM_009078, as a control) forward: 5′-CTGAAGGTCAAAGGGAATGTGTTC-3^′^, reverse: 5^′^-TTCGTGCTTCCTTGGTCT-TAGAC-3^′^, internal: 5^′^-TGCGAGCCTCAGCCTGGTCAGCC-3^′^. The first round of amplification used forward and reverse primers. The second round of amplification used one of the primers of the first round and a new internal primer. PCR reaction was performed in a 25-μl volume containing 12.5 μl iQ SYBR Green Supermix (Bio-Rad), 200 nM (in the first round) or 300 nM (in the second round) of each primer. In the first round of amplification, a 1 μl aliquot of the RT product was used and then 2.5 μl of the first-round product was used in the second round of amplification. Negative control samples were taken from the aspiration buffer without cells. The cDNA was amplified by real-time quantitative PCR with an ABI StepOnePlus Real-Time PCR System (Applied Biosystems, Foster City, CA). After 10 min of denaturation at 94°C, the amplification was performed with 20 (in the first round) or 40 (in the second round) thermal cycles of 94°C for 1 min, 56°C for 2 min, 72°C for 2 min, and a final extension at 72°C for 5 min. For quantification, Na_v_1.7 gene was normalized to the expressed housekeeping gene RPL19. The data were analyzed by the 2^-∆∆Ct^ method [40].

### Data analysis

All data are presented as means ± SEM. SigmaStat 3.5 was used for data analysis. A one-way ANOVA, with a Bonferroni procedure for post hoc was used in comparisons of cell membrane properties, Na^+^ currents, and mRNA and protein of Na_v_ 1.7. All data were confirmed by the Kolmogorov-Smirnov test to fit reasonably within normal distribution and equal variance was confirmed by the Levene test. Statistical significance was accepted when *P* < 0.05.

## Abbreviations

DMEM: Dulbecco’s modified Eagle’s medium; dibutyryl cyclic AMP: N^6^,2^′^-O-dibutyryladenosine 3^′^,5^′^-cyclic monophosphate; SDS: Sodium dodecyl sulfate; PVDF: Polyvinylidene fluoride; TEA: Tetraethylammonium; 4-AP: 4-aminopyridine; TTX: Tetrodotoxin.

## Competing interests

The authors declare that they have no competing interests.

## Authors’ contributions

YLL was principal investigator of the study. JL and YLL designed the research protocol. JL, HT, DZ, and HZ performed experiments. JL, HT, DZ, HZ, and YLL analyzed and interpreted results of experiments. JL and YLL drafted and edited manuscript. All authors read and approved final version of manuscript.
